# Pulmonary Artery Dissection: A Fatal Complication of Pulmonary Hypertension

**DOI:** 10.1155/2016/4739803

**Published:** 2016-11-15

**Authors:** Chuanchen Zhang, Xiaoyong Huang, Shuhua Li, Hengchen Yao, Bin Zhang

**Affiliations:** ^1^Department of Radiology, Liaocheng People's Hospital, Shandong University, 67 Dongchang West Road, Liaocheng District, Shandong 252000, China; ^2^Department of Radiology, Anzhen Hospital, Capital Medical University, 2 Anzhen Road, Chaoyang District, Beijing 100029, China; ^3^Department of Cardiology, Liaocheng People's Hospital, Shandong University, 67 Dongchang West Road, Liaocheng District, Shandong 252000, China; ^4^Department of Surgery, Liaocheng People's Hospital, Shandong University, 67 Dongchang West Road, Liaocheng District, Shandong 252000, China

## Abstract

Pulmonary artery dissection is extremely rare but it is a really life-threatening condition when it happens. Most patients die suddenly from major bleeding or tamponade caused by direct rupture into mediastinum or retrograde into the pericardial sac. What we are reporting is a rare case of a 46-year-old female patient whose pulmonary artery dissection involves both the pulmonary valve and right pulmonary artery. The patient had acute chest pain and severe dyspnea, and the diagnosis of pulmonary artery dissection was confirmed by ultrasonography and CT angiography. Moreover, its etiology, clinical manifestations, and management are also discussed in this article.

## 1. Introduction

Pulmonary artery dissection is an exceptionally rare and deadly condition, usually paired with pulmonary hypertension [1, 2]. Unlike aortic dissection, pulmonary artery dissection progresses rapidly and typically ruptures rather than developing a reentry site, which causes cardiogenic shock or sudden death [2–4]. It is thus mainly involved in the pulmonary artery trunk, without involvement of its branches or pulmonary valve [4, 5]. Furthermore, pulmonary artery dissection involving both the pulmonary valve and right pulmonary artery has never been reported.

In this article, there is presentation of the first pulmonary artery dissection case including both the pulmonary valve and right pulmonary artery with a patent ductus arteriosus (PDA). We discuss specific ultrasound and multidetector computed tomography (CT) angiography findings and briefly review the etiology, clinical manifestations, and management.

## 2. Case Report

With chest pain and shortness of breath, a 46-year-old woman was admitted to the emergency department. One day before her admission, her symptoms developed into acute chest pain and severe dyspnea during physical exertion. Her symptoms were described as severe, substernal pressure radiating into the shoulder associated with shortness of breath and nausea. At the age of forty one, the patient suffered dyspnea and edema in the lower limbs (New York Heart Association Functional Class IV). She was admitted to a regional hospital center and was diagnosed with severe pulmonary hypertension secondary to a patent ductus arteriosus (PDA). Because of high mortality of anesthesia, surgical intervention was not suggested. Thereafter, the patient was treated with a long-term medical regimen of diuretics and digoxin.

Generic approval was given by the Ethics Committee at our institution for retrospective analysis of clinical and imaging data (the chairperson of the ethics committee: Guozhang Liu, the protocol number: 2014110802, and the date of approval: Nov 8, 2014). The patient's husband provided permission to publish these features of this case.

A physical examination revealed a regular heart rate of 90 beats min^−1^, a respiratory rate of 25 breaths min^−1^, and blood pressure of 95/60 mmHg. She was afebrile, with elevated jugular venous pressure. Cardiac auscultation revealed a continuous murmur located at the upper left sternal border and a loud pulmonary component sound from the second heart sound, heard loudest over the left sternal edge.

**Figure 1:**
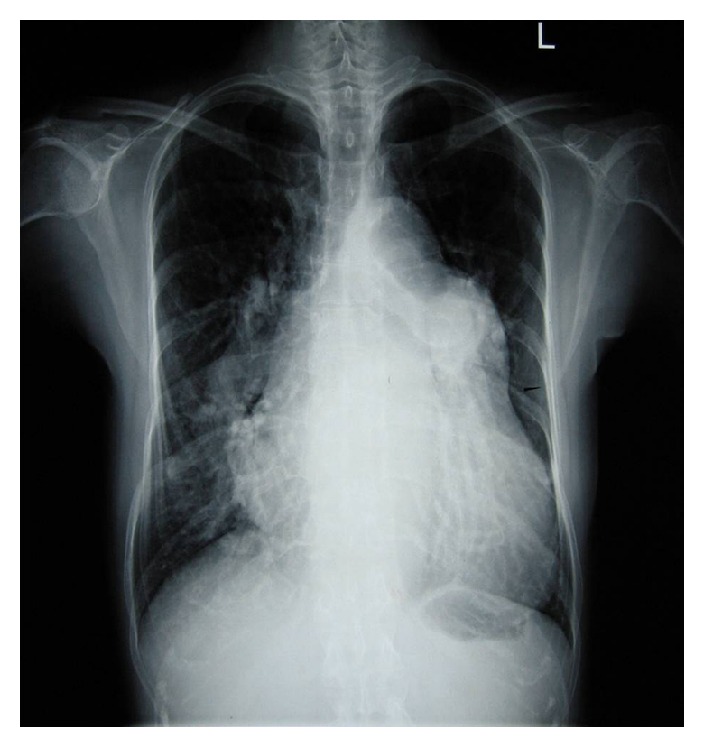
Plain radiographic features of pulmonary artery dissection. Posteroanterior chest radiograph showing cardiac enlargement and pulmonary arterial overcirculation. Pulmonary arterial overcirculation is indicated by prominent pulmonary arterial segments and greatly dilated hilar vessels.

The laboratory analyses revealed mild anemia (hemoglobin 98 g/L), hyponatremia (serum sodium 122 mmol/L), and elevated blood urea nitrogen (10.97 mmol/L). Her ECG showed sinus rhythm, right atrial enlargement, biventricular hypertrophy, and right axis deviation. A chest radiograph revealed moderate cardiomegaly and enormous dilatation of the pulmonary artery and its branches. The lungs and pleura were normal (Figure 1).

**Figure 2:**
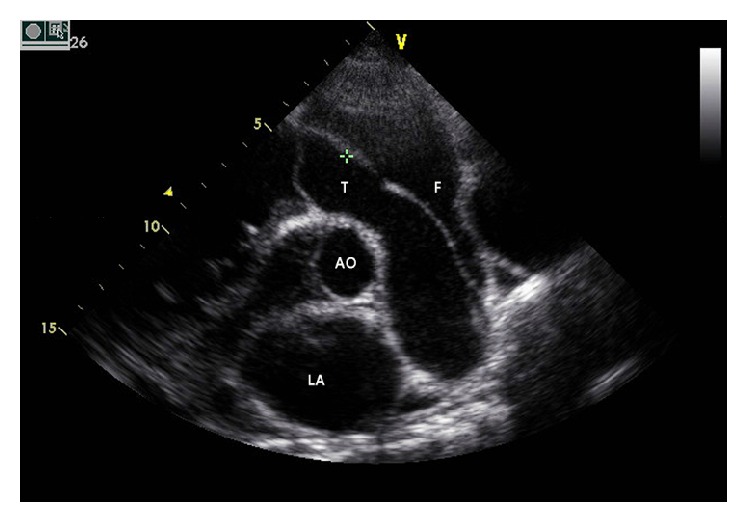
Echocardiogram. Two-dimensional transthoracic echocardiography in short-axis view of the heart base showing a greatly dilated pulmonary trunk with the dissection membrane (+). AO, aorta; LA, left atrium; T, true lumen; F, false lumen.

**Figure 3:**
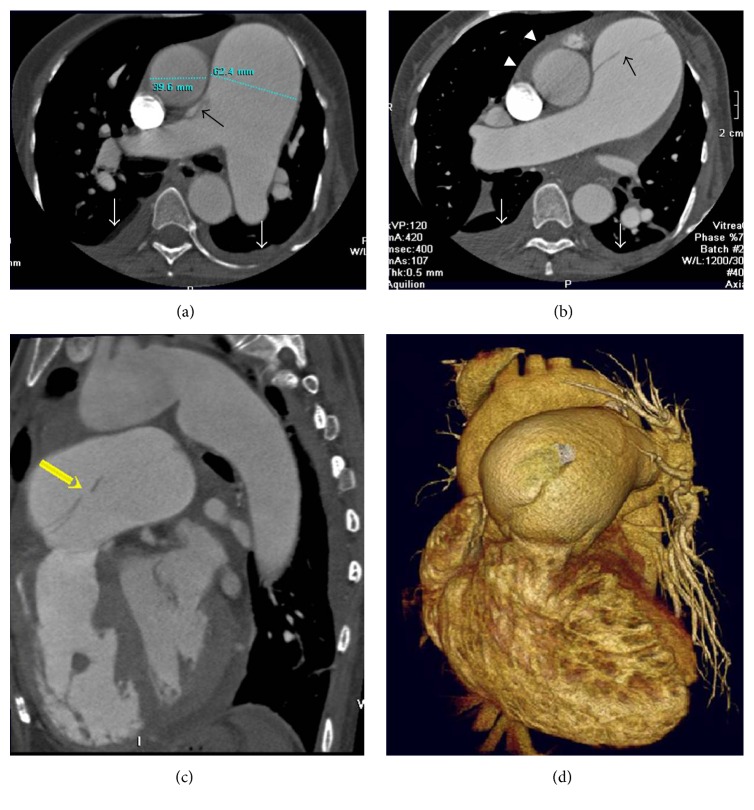
Intimal flap features determined on CT angiography. Transverse sections of CT pulmonary angiography (a, b) showing dilated central pulmonary arteries with an intimal flap (black arrow) in the main pulmonary trunk and right pulmonary artery. Pericardial effusion (white arrowheads) and bilateral pleural effusion (white arrows) are also noted. Sagittal MPR reconstruction (c) revealed the primary entry tear (arrow) in the proximal main pulmonary trunk and retrograde extension of the intimal flap to the pulmonary valve. Volume rendered images (d) vividly demonstrated the relationship between the intimal flap and pulmonary artery root.

Echocardiography showed enlarged right heart chambers, a greatly dilated main pulmonary trunk 64 mm in diameter with severe pulmonary regurgitation, as well as a PDA measured at 16 mm. We noted a dissection flap within the main pulmonary trunk (Figure 2) and performed an ECG-gated pulmonary CT angiography for further evaluation with a 64-slice CT scanner. CT axial images demonstrated marked dilation of the main pulmonary trunk (62.4 mm in diameter) and its central branches (Figure 3(a)). The intimal flap in the main and right pulmonary arteries was depicted as a dark line separating the true and false lumen (Figures 3(a) and 3(b)). Multiplanar reconstruction (MPR) clearly showed that the flap extended retrograde to the pulmonary valve from the inciting intimal tear in the proximal main pulmonary trunk (Figure 3(c)). Three-dimensional volume rendered (VR) images more validly demonstrated the relationship between the pulmonary valve and intimal flap (Figure 3(d)).

Combined lung and heart transplantations were proposed for this patient. Unfortunately, she suddenly collapsed and died 8 days after the onset of acute chest pain, before surgical intervention was possible. Her family declined an autopsy as the patient's diagnosis was clearly known.

## 3. Discussion

The diagnosis of pulmonary artery dissection is usually made in postmortem studies because it progresses rapidly and tends to rupture, causing cardiogenic shock or sudden death. Only 26 cases were documented in living patients so far since it was first described by Helmbrechtr in 1842 [4–8]. A review by Inayama et al. [5] indicated that the main pulmonary trunk is the dissection site in 72% of cases, followed by the intrapulmonary arteries (10%), the trunk and right main artery (6%), the left main artery (6%), the right main artery (4%), and the trunk plus both main arteries (2%).

The initiating event of artery dissection is known to be a tear through the intima into the mid or deep media, causing propagation of the dissection along intramedia planes. Pressurized blood in the media dissects the media longitudinally between concentric layers of elastin sheets, forming the false lumen. Even in aorta which is notable for thick media dominated by layers of elastin sheets, the dissection propagates more anterogradely than retrogradely with poorly understood reasons. The media of pulmonary artery are much thinner compared with that of aorta due to lower resistance to flow in the lung. Therefore, in pulmonary dissection, the false lumen tends to rupture rather than to develop a reentry site, as is usual in aortic dissection [6]. Interestingly, despite the dissection extents to the distal right pulmonary artery, her proximal pulmonary valve was also involved.

Pulmonary artery dissection occurs in both sexes with a slight female predominance (male : female 1 : 1.2). Patient ages ranged from 26 days to 85 years with incidence peaks occurring in the third and sixth decades [5, 6]. The etiology of pulmonary artery dissection remains unclear because few cases have been reported so far. Postmortem findings indicated the majority of pulmonary artery dissections occur in the presence of medial degeneration with fragmentation of elastic fibres and generalized dilatation of the pulmonary arterial tree. Pulmonary artery dissections are normally caused by chronic pulmonary hypertension and secondary to congenital cardiovascular anomalies, acquired valvular diseases, altered perfusion ventilation in COPD, or primary pulmonary hypertension [7–10], while minority of pulmonary artery dissections occur in aneurysm which associated with congenital lesions, infection, trauma, atherosclerosis or connective tissue disorders [11, 12].

Pulmonary artery dissection symptoms are nonspecific, and 82% of patients have exertional dyspnea, 67% have retrosternal chest pain, and 52% have central cyanosis [6, 12]. Pulmonary artery dissection is suspected in the face of sudden hemodynamic decomposition in an otherwise previously stable patient with known presentation of pulmonary hypertension or pulmonary artery aneurysm [13]. The condition progresses rapidly, so prompt diagnosis permits surgical treatment, which improves prognosis [14, 15]. Fortunately, several imaging modalities, such as chest radiograph, echocardiography, CT angiography, MRI, and cardiac catheterization, have been available for the evaluation of pulmonary artery dissection.

Chest radiograph is a commonly used imaging method to evaluate chest pain patients. Unfortunately, chest radiographs are abnormal in almost all pulmonary artery dissection cases but nonspecific. Common radiographic abnormalities include cardiomegaly, central pulmonary artery dilatation, and pleural effusion [16]. Chest radiographic findings may indicate if echocardiography or CT angiography should be the next evaluation method.

Khattar et al. [10] suggested ultrasonography should be performed first due to its ready accessibility, ease of use, and low cost and the fact that it is very sensitive at detecting main pulmonary artery dissection. However, for the left and right main pulmonary arteries or peripheral artery dissection, intimal flaps might not be readily identified by ultrasonography. Pulmonary CT angiography can show pulmonary artery dissection directly, as does pulmonary angiography. It is also noninvasive, cheaper, and widely available [17]. The segmented and subsegmented vessels are better demonstrated and findings are easier to interpret. CT scanning is the only test that can provide significant additional information related to alternate diagnoses such as pulmonary embolism (PE), which is important in an acute setting. Also, abundant postprocessing CT techniques make pulmonary artery dissection diagnosis more accurate and convenient.

Although investigators have reported the feasibility of MRI and conventional angiography at evaluating pulmonary artery dissection, MRIs are mostly limited to the evaluation of patients who have renal dysfunction or other contraindications for the use of iodinated contrast material, and conventional angiography is limited to patients in whom other results are nondiagnostic (clinical suspicion is high) [13, 18].

Several factors determine the best modality for the initial evaluation and postoperative follow-up of pulmonary artery dissection. These factors include the patient's condition stability, renal function, suspected postoperative complication, and each imaging modality's availability. Although the intimal flap can be detected by echocardiography, CT, MRI, and catheter angiography, we believe that echocardiography should be the first choice to evaluate the cause of chest pain or shortness of breath which is not only to look for pulmonary artery dissection but also to exclude other causes of chest pain, assess the valves, measure the PA pressure, and further assess underlying cardiovascular pathophysiology and haemodynamics. In the emergency setting, echocardiography will also be the most readily available test and the easiest way to apply in a bedside manner. Nevertheless, CT or MR pulmonary angiography could provide additional or confirmatory information. There is no definite guideline as the optimal treatment for pulmonary artery dissection. Surgical intervention may be necessary for most patients but it is usually followed by fatal consequences [12, 14]. Unfortunately, this patient suddenly died before surgical intervention.

In summary, although pulmonary artery dissection is an extremely rare condition, it should be suspected in patients with chronic pulmonary arterial hypertension presenting with chest pain, worsening dyspnea, or hemodynamic instability. Physicians should familiarize themselves with clinical features and imaging modalities of this condition so that a definitive diagnosis can be made and a proper treatment can be carried out.
